# Anoctamin 1/TMEM16A controls intestinal Cl^−^ secretion induced by carbachol and cholera toxin

**DOI:** 10.1038/s12276-019-0287-2

**Published:** 2019-08-05

**Authors:** Byeongjun Lee, Gyu-Sang Hong, Sung Hoon Lee, Hyungsup Kim, Ajung Kim, Eun Mi Hwang, Jiyoon Kim, Min Goo Lee, Jin-Young Yang, Mi-Na Kweon, Chung-Ming Tse, Donowitz Mark, Uhtaek Oh

**Affiliations:** 1 College of Pharmacy, Seoul National University, Seoul, 08826 Korea; 2 Brain Science Institute, Korea Institute of Science & Technology (KIST), Seoul, 02792 Korea; 3 College of Pharmacy, Chung-Ang University, Seoul, 06974 Korea; 4 Department of Pharmacology, Yonsei University College of Medicine, Seoul, 03722 Korea; 5 Mucosal Immunology Laboratory, Department of Convergence Medicine, University of Ulsan College of Medicine/Asan Medical Center, Seoul, 05505 Korea; 6 Departments of Physiology and Medicine, Division of Gastroenterois maintained by the opening of plasmalogy, Johns Hopkins University School of Medicine, Baltimore, MD USA

**Keywords:** Pathogenesis, Physiology, Pathogenesis, Physiology

## Abstract

Calcium-activated chloride channels (CaCCs) mediate numerous physiological functions and are best known for the transport of electrolytes and water in epithelia. In the intestine, CaCC currents are considered necessary for the secretion of fluid to protect the intestinal epithelium. Although genetic ablation of ANO1/TMEM16A, a gene encoding a CaCC, reduces the carbachol-induced secretion of intestinal fluid, its mechanism of action is still unknown. Here, we confirm that ANO1 is essential for the secretion of intestinal fluid. Carbachol-induced transepithelial currents were reduced in the proximal colon of *Ano1-*deficient mice. Surprisingly, cholera toxin-induced and cAMP-induced fluid secretion, believed to be mediated by CFTR, were also significantly reduced in the intestine of *Ano1*-deficient mice. ANO1 is largely expressed in the apical membranes of intestines, as predicted for CaCCs. The *Ano1*-deficient colons became edematous under basal conditions and had a greater susceptibility to dextran sodium sulfate-induced colitis. However, *Ano1* depletion failed to affect tumor development in a model of colorectal cancer. We thus conclude that ANO1 is necessary for cAMP- and carbachol-induced Cl^−^ secretion in the intestine, which is essential for the protection of the intestinal epithelium from colitis.

## Colitis: Intestinal membrane protein implicated in defective fluid secretion

An ion channel, a membrane protein allowing ion transport, that controls the flow of chloride is needed for proper secretion of protective fluids in the intestine. Uhtaek Oh from the Korea Institute of Science & Technology in Seoul, South Korea, and colleagues showed that cells lining the intestinal surface express a calcium-activated chloride channel called anoctamin-1 (ANO1) that regulates fluid secretion in the gut. Compared to control animals, ANO1-deficient mice released less fluid into their intestines following exposure to a diarrhea-inducing toxin or to a chloride transport–stimulating signaling molecule. This fluid secretion was previously thought to be mediated via a different ion channel. The ANO1-deficient mice accumulated fluid within colonic tissues, which increased their susceptibility to colitis. The findings point to ANO1 activation as a potential therapeutic strategy for treating colitis.

## Introduction

The secretion of intestinal fluid in the gastrointestinal tract is crucial for its normal physiological function^1^. Intestinal secretory processes provide an appropriate environment for digestion, and the secreted fluid protects the intestinal surface from dryness, bacterial infection, and physical damage as the food bolus passes through the intestinal tract^2,3^. Not surprisingly, dysfunction of intestinal fluid secretion contributes to intestinal infection, inflammation, and diarrhea and can also contribute to the intestinal effects of cystic fibrosis^1^. The primary contributors to the secretion of intestinal fluid are the anion secretory proteins that move Cl^–^ and HCO_3_^−^ across the enterocyte brush borders. The electrochemical Cl^−^ gradient is maintained by the uptake of Cl^−^ by basolateral Na–K–Cl cotransporter 1, and the intracellular electrogenic driving force is maintained by the opening of plasma membrane K^+^ channels^4^. Conditions that involve increased secretion of intestinal fluid result in diarrhea^1,5^.

Cystic fibrosis transmembrane conductance regulator (CFTR) is a Cl^−^ channel activated by intracellular cyclic adenosine monophosphate (cAMP) and adenosine triphosphate^6–8^ and is considered to be the major channel for Cl^–^ secretion in the intestine^5,9^. This conclusion is primarily based on the distal ileal obstruction experienced by patients with cystic fibrosis, particularly around the time of birth, which is believed to be a consequence of inadequate luminal hydration caused by the defective intestinal secretion of Cl^−10^. In addition, stimulation of intracellular cAMP by cholera toxin causes diarrhea, a drastic form of intestinal secretion^11–13^. However, another class of Cl^−^ channels, specifically, Ca^2+^-activated Cl^−^ channels (CaCCs), are also involved in the secretion of Cl^−^. CaCCs are activated by elevated levels of intracellular Ca^2+^ and mediate numerous physiological functions, including the transepithelial secretion of Cl^–^^4,14^. Carbachol activation of Cl^−^ conductance was demonstrated in colonic epithelial cells^15^. An increase in intracellular levels of Ca^2+^ evoked Cl^−^ currents in the T84 human colonic epithelial cell line^16^. Carbachol-induced Cl^−^ currents occur in the absence of CFTR^17^. CaCCs are also activated by rotavirus via the enterotoxin NSP4^18^. In addition, the lack of symptoms of cystic fibrosis in the intestines of some CFTR-deficient mice also suggests the presence of an alternative secretion pathway^17^.

Anoctamin 1 (ANO1; also known as transmembrane member 16A [TMEM16A]) is a Cl^−^ channel that is activated by intracellular Ca^2+^ and voltage and resembles a CaCC in its biophysical and pharmacological properties^19–21^. ANO1 has nine additional isoforms; these isoforms in the channel family are associated with several chronic diseases, such as gnathodiaphyseal dysplasia and Scott syndrome^22–27^. A functional role for ANO1 in intestinal secretion was previously suggested in a study in which genetic and pharmacological changes in ANO1 were induced. ANO1 is expressed in the distal colon^28,29^. Genetic disruption of ANO1 results in the loss of carbachol-induced transient Cl^−^ secretion in the neonatal colon^30^. A synthetic ANO1 antagonist inhibits the ATP-induced CaCC currents in T84 cells, a human colonic cell line^31^. In addition, Schreiber and colleagues found that the depletion of *Ano1* reduced the Ca^2+^-dependent secretion of Cl^−^ in the small and large intestines^32^. In addition, although these phenotypic studies suggest the involvement of CaCCs in Ca^2+^-induced Cl^−^ secretion, whether CaCCs mediate cAMP-induced Cl^−^ secretion is not known. In fact, cross-talk between the CFTR- and CaCC-signaling pathways has been reported. For example, isoproterenol and forskolin, which are known to stimulate adenylate cyclase, also increase the intracellular Ca^2+^ concentration^33,34^. Forskolin-induced Cl^−^ secretion was partially blocked by CaCC blockers such as 4,4′-diisothiocyanatostilbene-2,2′-disulphonic acid (DIDS) or 5-nitro-2-(3-phenylpropylamino)-benzoate (NPPB) in T84 cells^35^. In addition, cholera-toxin-induced fluid secretion occurred in intestines isolated from mice with CFTR deficiency, indicating the involvement of other chloride channels^36^. Thus, the role of CaCCs in mediating cAMP-induced Cl^−^ secretion in the intestines has been suggested but is largely unproven.

Thus, this study was performed to determine the functional role of ANO1 in fluid secretion in the intestine, with a special emphasis on cAMP-dependent fluid secretion. To achieve this objective, we generated two lines of conditional knockout (CKO) mice that have functionally disabled ANO1 in the small intestine and in the colon and observed the results of this genetic disruption of ANO1 in the gut. We found that ANO1 mediates Ca^2+^- and cAMP-dependent fluid secretion. In addition, we confirmed the expression of ANO1 in the apical membrane of the intestinal epithelium, consistent with the proposed mechanisms of action underlying CaCC-mediated intestinal fluid secretion.

## Materials and methods

### Generation of small intestine-specific and colon-specific Ano1 knockout mice

Floxed mice were generated as previously described^37^. These mice were crossed with *Villin*-*cre* (Jackson Laboratory, #004586) or *Cdx2-cre* mice (Jackson Laboratory, #009350). *Cdx2-Ano1*^fl/+^ or *Villin*-*Ano1*^fl/+^ mice were then crossed with *Ano1*^fl/+^ mice to generate *Ano1*^fl/fl^, *Cdx2*-*Ano1*^fl/fl^, or *Villin*-*Ano1*^fl/fl^ mice. These mice were used for experiments between 2 and 5 months of age.

### Animals

Two- to five-month-old male and female mice were used for experiments. Animal care and experiments were performed in accordance with the guidelines issued by the Institutional Animal Care and Use Committee of Seoul National University. Mice were housed with free access to food and water under a 12-h light/dark cycle.

### Immunofluorescence staining

Immunofluorescence staining of the jejunum and colon for ANO1 was performed as previously described^21^. Briefly, samples of both the proximal small intestine and colon from *Ano1*^fl/fl^ mice were sectioned with a cryostat (10-μm-thick sections) and incubated in methanol for 15 min at 4 °C. The samples were blocked with 4% bovine serum albumin in phosphate-buffered saline (PBS) with Tween 20 for 30 min and incubated with a rabbit polyclonal antibody specific for ANO1 (Abcam, ab53212, diluted 1:100, in blocking solution) overnight at 4 °C. The samples were washed and incubated with Alexa Fluor 594-conjugated donkey anti-rabbit (A21207, Molecular Probes, diluted 1:800 in blocking solution) for 1 h at room temperature. The samples were then washed and imaged with a Leica TCS confocal system (Leica Microsystems).

### Preparation of intestinal epithelial cells

Primary intestinal epithelial cells (IECs) were prepared with slight modification of a previous report^38,39^. The duodenum, jejunum, and ileum in the small intestine and the proximal and distal colon were removed from *Ano1*^fl/fl^ or *Cdx2*-*Ano1*^fl/fl^ mice. To invert the intestinal segments, each segment was inserted slightly into a glass rod that had a constriction immediately next to one smooth end. We tied the end of the intestine onto the glass rod and rolled the intestine over to the end to expose the mucosal surface. Everted intestinal segments were washed twice with ice-cold Mg^2+^ and Ca^2+^-free Hank’s balanced salt solution (HBSS) containing 0.5 mM dithiothreitol (Sigma). The tissues were suspended in 50 ml of HBSS wash solution and then vigorously inverted ten times. The tissues were transferred to a fresh 50 ml of HBSS wash solution and vigorously inverted an additional four times. After the removal of the supernatant, the tissues were incubated at 37 °C for 1 h with a digestion solution containing 200 U/mL collagenase type 1 (Sigma), hyaluronidase 100 U/ mL, and 1% fetal bovine serum in DMEM containing glucose and l-glutamine, without sodium pyruvate (Gibco). The tissues were centrifuged at 100×*g* for 5 min at 4 °C and washed with 2% fetal bovine serum in DMEM. The tissues were filtered through a 50-μm strainer with centrifugation at 200×*g* for 4 min at 4 °C. After the removal of the supernatant, the cells were ready for further analysis. For primary colonic epithelial cell culture, mouse colons were everted and washed with ice-cold Ca^2+^-free Dulbecco’s modified Eagle’s medium (DMEM). The samples were incubated in Ca^2+^-free DMEM for 5 min at 37 °C and with 20 mg of collagenase P (Roche) in Ca^2+^-free DMEM for an additional 10 min with shaking. The colon was removed, and the residual solution was centrifuged at 10 × g for 2 min at 4 °C. The supernatant was collected and centrifuged at 120 × g for 2 min at 4 °C. The pellets were replated in DMEM supplemented with 10% fetal bovine serum and penicillin–streptomycin. All of the experiments were performed within 12 h of animal sacrifice.

### Western blot analysis

Western blotting was performed as previously described^40^. Briefly, protein samples from intestinal epithelial cells of *Ano1*^fl/fl^ and *Cdx2*-*Ano1*^fl/fl^ mice were mixed with Laemmli buffer and separated by 8% sodium dodecyl sulfate-polyacrylamide gel electrophoresis. Migrated proteins on the gel were transferred to Immobilon-P polyvinylidenedifluoride membranes (Millipore) for 2 h on ice at 200 mA. The membranes were blocked with 2.5% skim milk in Tris-buffered saline and Tween 20 for 1 h and incubated with a rabbit polyclonal antibody specific for ANO1 (diluted 1:100, Abcam) overnight at 4 °C. The membranes were incubated for an additional 1 h at room temperature. A secondary horseradish peroxidase-conjugated anti-rabbit antibody was used at a 1:5000 dilution for 1 h at room temperature. The blots were visualized by chemiluminescence using LAS 4000 (GE Healthcare). β-actin (Santa Cruz, sc-47778) was used as a positive control. The relative protein amount was calculated as the chemiluminescence intensity of a protein blot divided by the intensity of the β-actin blot.

### Ussing chamber/voltage-clamp recording

Ussing chamber recording was performed as previously described^28^. Briefly, small pieces of colons were mounted in an Ussing chamber/voltage-clamp assembly (World Precision Instrument) with an aperture of 0.25 cm^2^. The colon was divided into two parts: the proximal colon was defined as the segment starting from the end of the cecum to the beginning of the proximal transverse colon, and the distal colon was defined as the segment from the end of the proximal transverse colon to the sigmoid colon. The standard Krebs–Henseleit solution contained 118 mM NaCl, 4.7 mM KCl, 23 mM NaHCO_3_, 1.2 mM K_2_HPO_4_, 1.2 mM CaCl_2_, 1.2 mM MgCl_2_, 0.3 mM ethylenediaminetetraacetic acid, and 10 mM glucose. The solution in the reservoirs was gassed with 5% CO_2_ and 95% O_2_. The solution was maintained at 37 °C by water jackets.

The short-circuit current (*I*_sc_) was measured with an automatic voltage-clamping device (World Precision Instruments) that compensates for the resistance of the solution between the voltage difference-measuring electrodes. The transepithelial potential (*V*_t_) was recorded through 3 M KCl-agar bridges connected to a pair of calomel half-cells. The transepithelial current was applied across the tissue via a pair of Ag/AgCl electrodes that were kept in contact with the mucosal and serosal bathing solution. All experiments were performed under short-circuited conditions. The tissue was placed in the apparatus and equilibrated for 1 h to stabilize the *I*_sc_ before the experiment began. The I_sc_ is negative when a positive current flows from the serosa to the mucosa. A positive *I*_sc_ corresponds to the net electrogenic secretion of anions or the net electrogenic absorption of cations. Transepithelial resistances (*R*_t_) were obtained by applying short current pulses (ΔI). *R*_t_ and *I*_sc_ were calculated with Ohm’s law (*I*_sc_ = *V*_t_/*R*_t_, *R*_t_ = Δ*V*_t_/ΔI). All *I*_sc_ values were normalized to μA/cm^2^ with the window area of the tissue slider. The baseline value of electrical parameters was determined as the mean over the 2 min immediately prior to drug administration. To block Na^+^ and K^+^ currents, 100 μM amiloride and 5 mM BaCl_2_ were added to the mucosal and serosal sides, respectively. Carbamoylcholine (1 mM) was used to stimulate Cl^−^ secretion. All experiments were performed under voltage-clamp conditions.

### Whole-cell current recording

Whole-cell recording was performed as previously described^21^. Isolated colonocytes from both genotypes were used for whole-cell current recordings. After a gigaseal was formed, the membrane patch below the pipette tip was ruptured. The junctional potentials were adjusted to zero. The pipette solution contained (in mM) 130 CsCl, 2 MgCl_2_, 10 CsOH/HEPES, 2 adenosine triphosphate, and 0.3 guanosine triphosphate. The solution was adjusted to pH 7.3 with the addition of HCl. The bath solution contained 140 mM NMDG, 10 mM HEPES, and 2 mM MgCl_2_ adjusted to pH 7.3 with the addition of HCl. The voltage-clamp recordings were performed with an Axopatch 200B amplifier (Molecular Devices). The data were digitized with a Digidata 1440 digitizer (Molecular Devices) and stored on a personal computer.

### Preparation of acute DSS-induced colitis and colorectal cancer

To induce acute DSS-induced colitis, three-month-old littermates received 2% DSS (MP Biomedicals) in their drinking water for 5 days and were then sacrificed. A colorectal cancer model was prepared as previously described^41^. An intraperitoneal injection of azoxymethane (10 mg/kg) was administered on day 1, and 2% DSS was given for 8 days *ab libitum*. PBS was then given for the next 14 days for recovery. Two more cycles were repeated, and the mice were sacrificed on day 60.

### Cytokine levels

The large intestines were opened longitudinally. Their contents were removed by shaking in cold PBS, and the tissues were cut into pieces 1- to 2-cm long. The intestinal epithelial cells and mucus were removed by shaking the tissues in ethylenediaminetetraacetic acid buffer (10 mM in PBS) for 30 min at 37 °C. After washing with prewarmed PBS, the tissues were chopped and incubated with complete medium for 24 h at 37 °C. The cytokine levels in the culture supernatant (data not shown) were measured using the Cytometric Bead Array-Mouse Inflammation Kit (BD Biosciences, San Jose, California) according to the manufacturer’s instructions.

### Fecal protein determination

Total proteins in feces were measured by a bicinchoninic acid protein assay kit (Thermo Scientific) according to the manufacturer’s instructions. Briefly, fresh feces were harvested and thoroughly mashed with sterile PBS. Bovine serum albumin standards and feces samples were diluted and mixed well with working reagents. After incubation at 37 °C for 30 min, the samples were measured at 562 nm using a spectrophotometer. A standard curve was plotted based on the values of each BSA standard, and the protein concentration of each feces sample was determined.

### Statistics

All of the data are expressed as the mean ± SEM. The means between two groups were tested for statistical significance using an unpaired two-tailed Student’s *t*-test. To compare differences among more than two groups, one-way ANOVA was used followed by Tukey’s post-hoc test. *P* < 0.05 was considered significant.

## Results

### Generation of *Cdx2-Ano1*^fl/fl^ mice

**Fig. 1:**
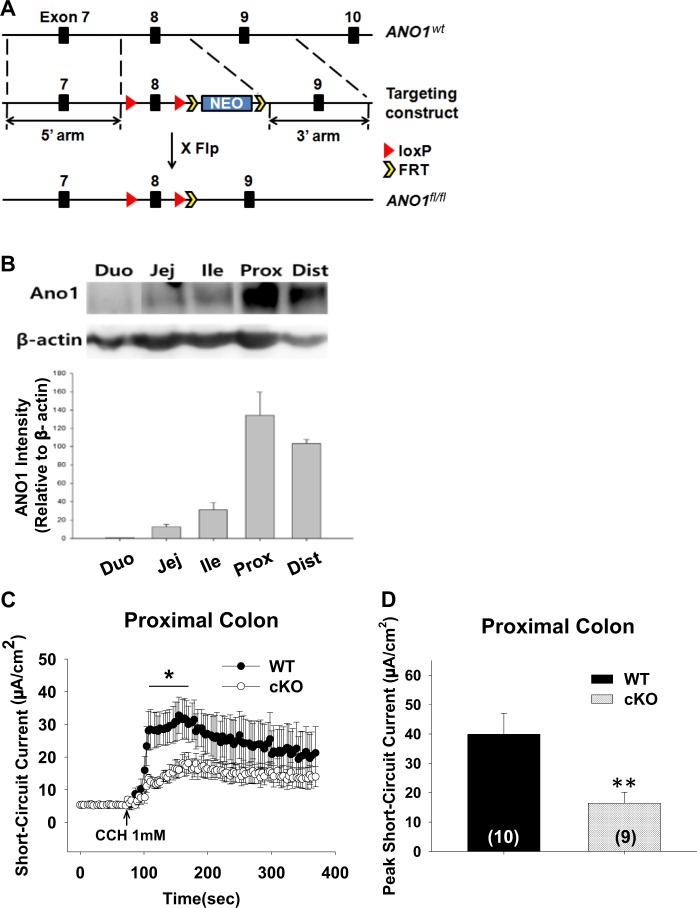
Ablation of *Ano1* from intestinal epithelial cells reduces carbachol-induced transepithelial Cl^−^ currents. **a** Schematic diagram for the construction of the *Ano1*^fl/fl^ mice. Exon 8 of *Ano1* was flanked by loxp sites. The *Ano1*^*fl/fl*^ mice were crossed with *Cdx2-cre* transgenic mice to obtain *Cdx2-Ano1*^fl/fl^ mice. **b** Western blots of ANO1 in intestinal epithelial cells (IECs) isolated from intestinal segments in mice. IECs were prepared from each segment of the small and large intestines. Actin is a loading control. The whole blot is shown in Supplementary Fig. 3. The experiments were repeated four times. duo; duodenum, jej; jejunum, ile; ileum, prox; proximal colon, dist; distal colon. **c** Changes in short-circuit currents evoked by 1 mM carbachol applied to the serosal surface of the colon isolated from *Ano1*^fl/fl^ (wild-type, WT) and *Cdx2-Ano1*^fl/fl^ (conditional knockout, CKO) mice. To block Na^+^ and K^+^ currents, 100 μM amiloride and 5 mM BaCl_2_ were added to the mucosal and serosal sides, respectively. **d** Summary of peak short-circuit currents evoked by carbachol applied to the colon of WT and *Cdx2-Ano1*^fl/fl^ (CKO) mice. **p < 0.01 and *p < 0.05. Error bars represent the standard error of the mean (SEM). The numbers in brackets represent the number of experiments

To specifically target the ANO1 protein in intestinal epithelial cells, we generated two different lines of mice that lacked ANO1 mainly in the small or large intestine. To target *Ano1* in the epithelial cells of the colon, *Ano1*-floxed (*Ano1*^fl/fl^) mice were crossed with *Cdx2-cre* transgenic mice to produce *Cdx2*-*Ano1*^fl/fl^ mice (Fig. 1a). *Cdx2* is a caudal-type homeobox gene and a transcriptional factor involved in the proliferation and differentiation of colonocytes that was used to create CKO mice in the colon^42^.

### Loss of carbachol-induced transepithelial Cl^−^ currents in the large intestine of *Cdx2-Ano1*^fl/fl^ mice

The expression profile in the intestines was determined by western blotting of segments of the small and large intestines. We isolated intestinal epithelial cells only from other tissues because ANO1 is rich in interstitial cells of Cajal embedded in mouse intestinal smooth muscles^43–46^. As shown in Fig. 1b, ANO1 is expressed in the small and large intestines. Most notably, ANO1 is richly expressed in intestinal epithelial cells of the colon.

To determine whether functional alterations were present in the epithelium of the colon in mice in which *Ano1* was knocked out, carbachol-induced transepithelial currents (short-circuit currents, *I*_sc_) of the proximal colon from control and *Cdx2*-*Ano1*^fl/fl^ mice were determined using the Ussing chamber/current clamp approach^28^. A rapid increase in *I*_sc_ occurred after the cholinergic agonist, 1 mM of carbachol^28,47^, was applied to the isolated colons from control mice (Fig. 1c). The carbachol-induced *I*_sc_ was significantly (*p* < 0.05; *n* = 9~10) reduced in the proximal segment of the colon in the *Cdx2*-*Ano1*^fl/fl^ mice (Fig. 1c, d).

### Loss of Ca^2+^ activated currents in colonocytes from *Cdx2-Ano1*^fl/fl^ mice

**Fig. 2:**
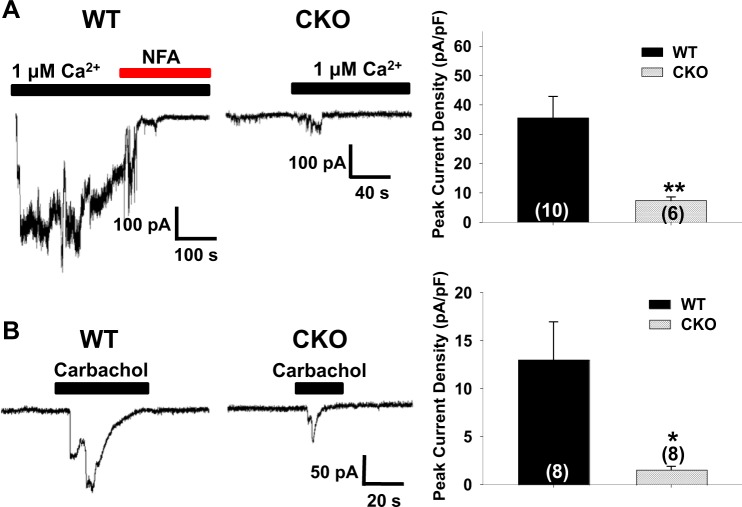
Genetic ablation of *Ano1* reduces Ca^2+^- and carbachol-induced Cl^−^ currents in colonocytes isolated from *Cdx2-Ano1*^fl/fl^ mice. **a** Traces of whole-cell Cl^−^ currents induced by Ca^2+^ in colonocytes isolated from *Ano1*^fl/fl^ (WT) and *Cdx2-Ano1*^fl/fl^ (CKO) mice. Ca^2+^ (1 μM) was added to the pipette. The pipette solution contained 140 mM CsCl. The bath solution contained 140 mM NMDG-Cl. The holding potential was −60 mV. Whole-cell patches were formed at the beginning of the black bar. Niflumic acid, a blocker of ANO1, was applied to block the ANO1 current. (Right panel) Summary of the peak current density evoked by Ca^2+^ in WT and CKO colonocytes. **p < 0.01. **b** Traces of whole-cell Cl^–^ currents induced by carbachol in colonocytes from WT and CKO mice. After forming whole-cell patches, 1 mM carbachol was applied to the bath. (Right) Summary of the current density evoked by carbachol in WT and CKO colonocytes. *p < 0.05

We next examined the CaCC currents in freshly isolated colonocytes from both genotypes. The anion currents were recorded from whole colonocytes after the application of 1 μM of Ca^2+^ to the pipette. The pipette and bath solutions contained 140 mM CsCl and NMDG-Cl, respectively. As soon as a whole-cell patch was formed, a large inward current was observed in the colonocytes isolated from the *Ano1*^fl/fl^ mice; this current was inhibited by 100 μM of niflumic acid, a CaCC blocker (Fig. 2a). These CaCC currents were found in 10 of 14 (72%) of the colonocytes in the *Ano1*^fl/fl^ mice. In contrast, the majority of the colonocytes (14 of 20; 70%) from the *Cdx2*-*Ano1*^fl/fl^ mice failed to show CaCC currents (Fig. 2a). Carbachol also evoked robust Cl^−^ currents, with average amplitudes of 13.0 ± 4.0 pA/pF in the majority of colonocytes (8 of 13) from the *Ano1*^fl/fl^ mice. In contrast, only 8 of the 21 colonocytes from the *Cdx2*-*Ano1*^fl/fl^ mice (38%) responded to the application of carbachol and had significantly reduced amplitudes (1.56 ± 0.36 pA/pF) (Fig. 2b).

### ANO1 is located in the apical membrane of jejunal and colonic epithelial cells

**Fig. 3:**
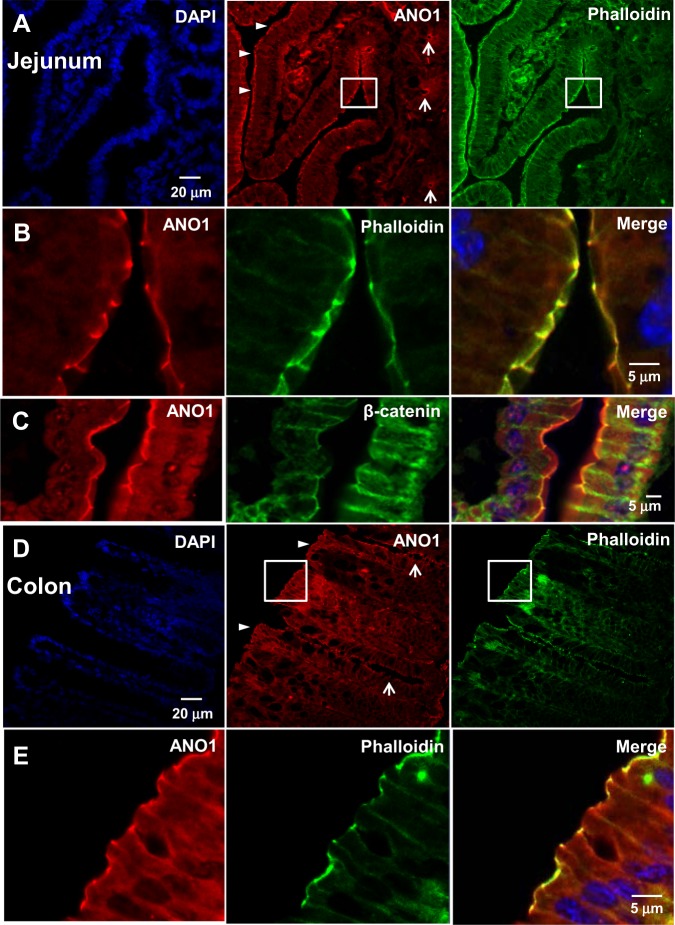
ANO1 is expressed in the apical membranes of the small intestine and colon. **a**–**e** Immunofluorescence images of the jejunum (**a**–**c**) and colon (**d**, **e**) stained with antibodies specific for ANO1, as well as phalloidin and β-catenin, markers for apical and lateral membranes, respectively. Arrowheads indicate the strong expression of ANO1 in the apical membrane of villi and surface epithelial cells in the jejunum (**a**) and colon (**d**). ANO1 showed relatively weak expression in the apical membrane of the crypt in the jejunum and colon (arrows) compared to the villi and surface region (arrowheads). **b**, **c**, **e** Magnified images of epithelial cells in the square regions of (**a**) and (**d**). ANO1 was largely colocalized with phalloidin at the apical membrane (**b**) but was not colocalized with β-catenin, which is the lateral membrane marker (**c**)

ANO1 immunoreactivity was found largely at the apical membrane of surface epithelial cells in the jejunum and proximal colon (Fig. 3). When costaining was performed with phalloidin, a marker of the apical membrane^28^, ANO1 colocalized with phalloidin (Fig. 3b, d, e). In contrast, when staining with β-catenin, a marker of the lateral membrane of gut epithelial cells^48^, ANO1 rarely colocalized with β-catenin (Fig. 3c). These results thus suggest that ANO1 is present at the apical surface of jejunal and colonic epithelial cells.

**Fig. 4:**
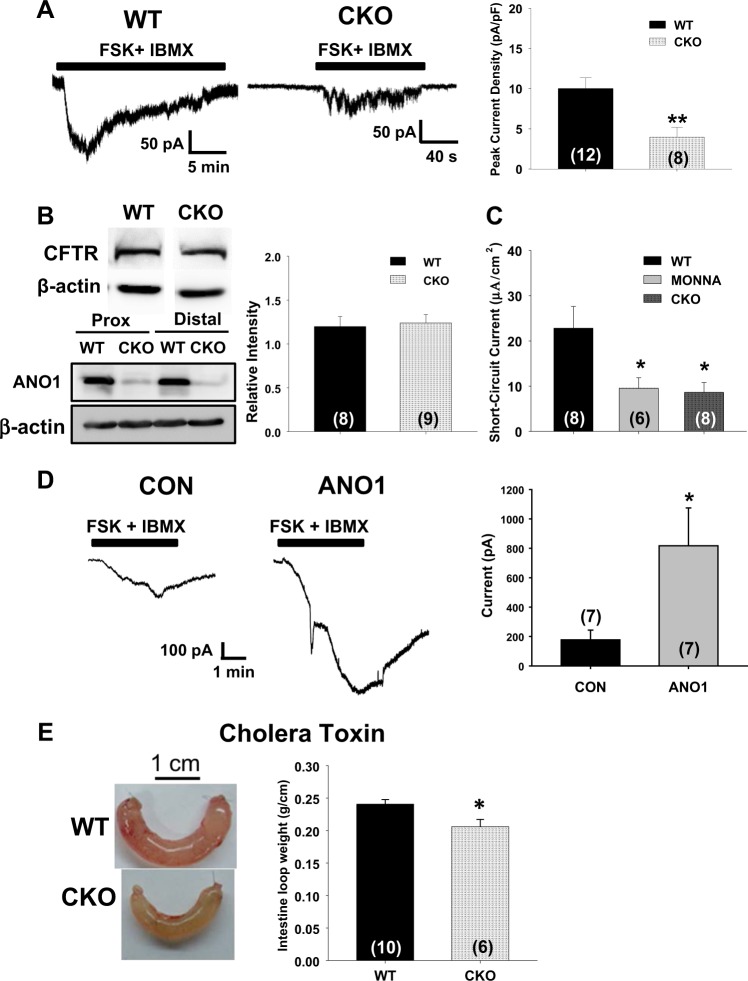
cAMP-dependent Cl^−^ and fluid secretion is reduced in the *Ano1*-deficient intestine. **a** Whole-cell Cl^–^ currents activated by the application of 5 μM forskolin (FSK) and 1 mM IBMX to colonocytes of the proximal colon isolated from *Ano1*^fl/fl^ (WT) and *Cdx2-Ano1*^fl/fl^ (CKO) mice. (Right) Summary of the peak current responses of colonocytes from both genotypes of mice after the application of FSK and IBMX. **p < 0.01. **b** Western blot of CFTR (~160 kDa) in colonocytes isolated from WT and CKO mice (upper panel) and ANO1 in the proximal and distal colon of both genotypes (lower panel). β-actin was investigated as a control. (Right) Summary of the intensities of blots performed with a CFTR antibody for colonocytes from both genotypes. The intensities of CFTR were normalized to that of β-actin. Whole blots of ANO1 and CFTR are shown in Supplementary Fig. 3. **c** Peak short-circuit currents (*I*_sc_) of proximal colons from WT and CKO mice induced by the application of FSK and IBMX to the serosal surface. The ANO1 blocker MONNA (100 µM) was added to the FSK- and IBMX-containing bath solution for the colons of WT mice. **d** Traces of whole-cell Cl^−^ currents induced by FSK and IBMX in mock (control vector) or ANO1-transfected H293T cells. (Right) Summary of FSK and IBMX evoked Cl^−^ currents in mock or ANO1-transfected H293T cells. **e** Fluid secretion induced by cholera toxin from a loop of jejunum isolated from WT and *Villin-Ano1*^fl/fl^ (CKO) mice. An intestinal loop was made at the jejunum after tying both ends approximately 4 cm apart. Cholera toxin (3 μg) in 100 μl of saline solution was injected into the intrajejunal loop. Six hours after injection, the intestinal loop was isolated, and its weight and length were measured. The ratio of weight (g) to length (cm) was considered an indication of fluid secretion. (Right) Summary of the intestinal loops of WT and CKO mice. *p < 0.05

### cAMP-dependent Cl^–^ secretion is reduced in the colon of *Cdx2-Ano1*^fl/fl^ mice

We then sought to determine whether the genetic disruption of *Ano1* in the intestine affects the cAMP-dependent secretion of Cl^–^, which is known to be mediated by CFTR^49^. To increase the intracellular levels of cAMP, 5 μM forskolin and 1 mM 3-isobutyl-1-methylxanthine (IBMX), an adenyl cyclase activator and a phosphodiesterase inhibitor, respectively, were applied to the isolated colonocytes. As shown in Fig. 4a, the application of 5 μM forskolin and 1 mM IBMX readily evoked robust Cl^−^ currents in colonocytes from the *Ano1*^fl/fl^ mice. Surprisingly, the Cl^−^ currents of the colonocytes evoked by forskolin and IBMX from the *Cdx2*-*Ano1*^fl/fl^ mice were significantly (*p* < 0.01; *n* = 8–12) smaller than those of the colonocytes from the *Ano1*^fl/fl^ mice (Fig. 4a), which suggests a reduction in cAMP-stimulated channel activities in the colon of the *Cdx2*-*Ano1*^fl/fl^ mice. Notably, the ablation of *Ano1* in the colon did not affect the expression level of CFTR, whereas it blocked the expression of ANO1 (Fig. 4b). Thus, the reduction in the Cl^−^ currents evoked by forskolin and IBMX that was observed with the colonocytes of the *Cdx2*-*Ano1*^fl/fl^ mice may be caused by a CFTR-independent pathway. Because the application of forskolin and IBMX to epithelial cells can elevate the intracellular levels of Ca^2+^ as well as cAMP^35,50^, it is highly likely that the application of forskolin and IBMX activates ANO1 in addition to CFTR. Indeed, the transepithelial Cl^−^ currents (*I*_sc_) evoked by forskolin and IBMX in the proximal colons of the control mice were significantly (*p* < 0.05; *n* = 6–8) reduced by the ANO1-specific blocker MONNA^51^, to a level comparable to that found in the *Cdx2*-*Ano1*^fl/fl^ mice (Fig. 4c). In addition, forskolin and IBMX were applied to control and Ano1-transfected HEK293T cells to determine if the cAMP pathway activates ANO1. Indeed, the application of 5 μM forskolin and 1 mM IBMX evoked robust Cl^−^ currents (817.3 ± 257.3 pA, *n* = 7) in ANO1-transfected H293T cells compared to those in mock-transfected cells (177.6 ± 66.4 pA, *p* < 0.05, *n* = 7) (Fig. 4d). These results clearly suggest that stimulation of the cAMP pathway activates ANO1.

### Cholera toxin-induced fluid accumulation is reduced in Ano1-deficient intestines

We then determined whether a sustained increase in the level of intracellular cAMP utilizes ANO1 to produce prolonged secretion of Cl^−^. Cholera toxin was used because it causes a prolonged increase in intestinal cAMP^52^. Jejunal loops were prepared from intestines of both genotypes and injected with 3 μg of cholera toxin in 0.1 ml of Ringer solution. After 6 h of incubation, the fluid accumulation in the loop was measured. To target ANO1 specifically in the small intestine, *Ano1*-floxed (*Ano1*^fl/fl^) mice were crossed with *Villin*-*cre* transgenic mice to produce *Villin-Ano1*^fl/fl^ mice. Villin is an actin-binding protein that is expressed in the brush borders of epithelial cells from the small intestine and colon^53^. As shown in Fig. 4e, the cholera toxin-induced fluid accumulation was slightly but significantly (~15%) lower in the jejunum of the *Villin*-*Ano1*^fl/fl^ mice than in that of the control mice. These results further suggest that an increase in the level of intracellular cAMP can activate ANO1 along with CFTR.

### Mild edema in the *Cdx2-Ano1*^fl/fl^ colon

**Fig. 5:**
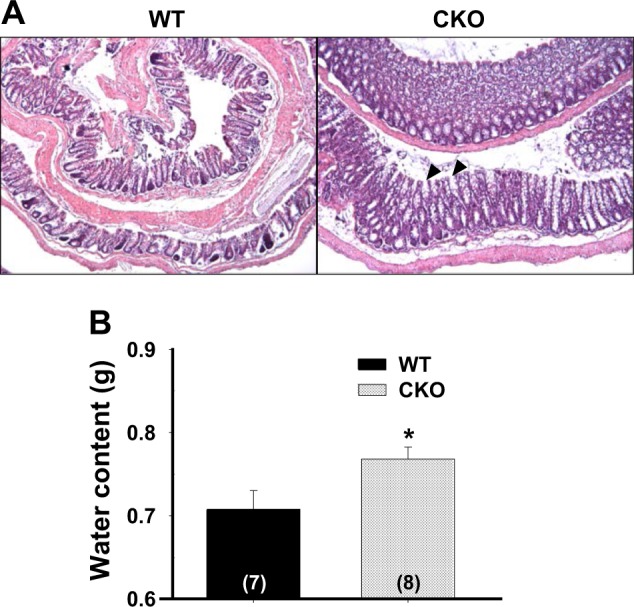
Colons of *Cdx2-Ano1*^fl/fl^ mice in steady state depict mild edema. **a** Hematoxylin and eosin staining of colons from *Ano1*^fl/fl^ (WT) and *Cdx2-Ano1*^fl/fl^ (CKO) mice (Swiss roll preparation). There was marked thickening of the mucosal layer (arrowheads) and submucosal edema. **b** Water content in the stool was significantly increased in the CKO mice. The water content in the stool was measured by dividing the wet weight minus the dry weight by the wet weight of the stool. *p < 0.05. Error bars represent the SEM. The numbers in brackets represent the number of experiments

Histologic examination revealed mucosal thickening and submucosal edema in the colons of the *Cdx2*-*Ano1*^fl/fl^ mice compared to those of the *Ano1*^fl/fl^ mice (Fig. 5a). There was also mild shedding of the epithelium of the mucosal layer (Supplementary Fig. 1). However, we failed to find ulcers in the epithelial lining of the colon from *Cdx2*-*Ano1*^fl/fl^ mice. In addition, the water content in the stool of *Cdx2-Ano1*^fl/fl^ mice was significantly (*p* < 0.05; *n* = 7–8) increased, suggesting diarrhea (Fig. 5b). Thus, these results suggest that mild edema prevails in the colon even under normal conditions if *Ano1* is deleted.

### Augmented sensitivity to inflammation in the *Cdx2-Ano1*^fl/fl^ colon

**Fig. 6:**
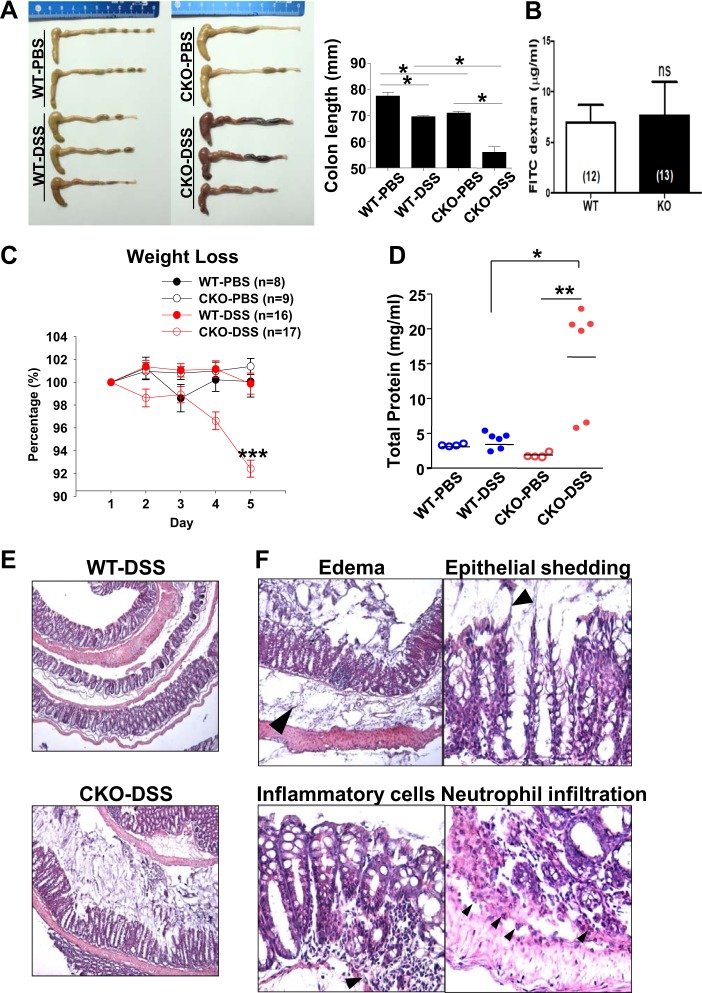
Acute treatment with dextran sodium sulfate induces severe colitis in CKO mice. **a** Colons isolated from *Ano1*^fl/fl^ (WT) and *Cdx2-Ano1*^fl/fl^ (CKO) mice were treated orally with phosphate-buffered saline (PBS) or 2% DSS-containing saline solution for 5 days. A greater reduction in the length of the colons from the CKO mice was observed after treatment with DSS. (Right) Summary of the colon lengths from both genotypes after treatment with PBS or DSS. *p < 0.05. **b** The barrier functions of the guts of WT and *Cdx2-Ano1*^*fl/fl*^ (KO) mice. The barrier function was determined by the dextran permeability through the gut. ns, not significant. **c** Changes in body weights of WT and CKO mice treated with PBS or DSS from day 1 to day 5. ***p < 0.001 compared to WT mice treated with DSS. **d** Total amount of protein extracted from feces collected from both genotypes on the fifth day after treatment with PBS or DSS. Horizontal bars represent the means. *p < 0.05, **p < 0.01. **e** Hematoxylin and eosin-stained colonic sections of *Ano1*^fl/fl^ (WT) and *Cdx2-Ano1*^fl/fl^ (CKO) mice treated with DSS. **f** Magnified sections of colons from CKO mice stained with hematoxylin and eosin. The submucosal layer was edematous (arrowhead), the epithelium was irregular (arrowhead) because of shedding, mononuclear cells (arrowhead) were prominent, and neutrophils (arrowhead) had infiltrated the submucosal layer in colons from CKO mice

If the secretion of Cl^–^ is essential for the protection of the epithelia in the gut, its genetic disruption would be expected to have pathologic consequences. To test this hypothesis, acute colitis was induced in control and *Cdx2*-*Ano1*^fl/fl^ mice by means of a 5-day oral treatment with 2% dextran sodium sulfate (DSS). Upon treatment with DSS, the colons of the *Cdx2*-*Ano1*^fl/fl^ mice became much shorter than those of the control mice (Fig. 6a).

Colitis might be the result of impaired barrier function. Therefore, we compared the barrier functions of both genotypes. The barrier function was determined by dextran permeability through the gut. After a 5-day oral treatment with 2% DDS, mice received 100 μl of FITC-dextran by oral gavage in sterile phosphate-buffered saline. Serum was collected 4 h later, and FITC levels in serum were measured. As shown in Fig. 6b, the level of FITC-dextran in the serum from *Villin-Ano1*^fl/fl^ mice was not different from that of the WT mice. Therefore, the barrier function of the gut in *Ano1*-ablated mice is comparable to that in the WT mice.

The body weights of the *Cdx2*-*Ano1*^fl/fl^ mice were drastically reduced compared with those of the *Ano1*^fl/fl^ mice from 4 days after treatment with DSS (Fig. 6c). The total protein level in the fecal extracts was much higher in the DSS-treated *Cdx2*-*Ano1*^fl/fl^ mice than in the control groups (*p* < 0.05, one-way ANOVA; Fig. 6d).

Histologic examination of the colons of *Cdx2*-*Ano1*^fl/fl^ mice after treatment with DSS revealed severe epithelial shedding and edema (Fig. 6e, f and Supplementary Fig. 1a). The submucosal layer became severely edematous (Fig. 6f). Irregular and distorted folds of epithelia were observed, consistent with epithelial cell shedding (Fig. 6f). Infiltration of mononuclear cells and neutrophils was prominent in the submucosa of the colon from the DSS-treated *Cdx2*-*Ano1*^fl/fl^ mice. No significant infiltration of neutrophils was observed in the control mice (Fig. 6e). In addition, apoptotic cell death was more evident in the DSS-treated colons from the *Cdx2*-*Ano1*^fl/fl^ mice than in those from control mice by terminal deoxynucleotidyl transferase dUTP nick end labeling (Supplementary Fig. 1b). However, the levels of proinflammatory cytokines, including γ-interferon, tumor necrosis factor α, interleukins 6 and 10, interleukin 12 p70, and monocyte chemotactic protein 1, in the culture supernatant of the colon homogenates were not different between the colons of the two genotypes (Supplementary Fig. 2). Taken together, these results suggest that the *Cdx2*-*Ano1*^fl/fl^ mice showed increased susceptibility to DSS-induced inflammation in the colon.

### Lack of susceptibility to colorectal cancer in *Cdx2-Ano1*^fl/f^ mice

**Fig. 7:**
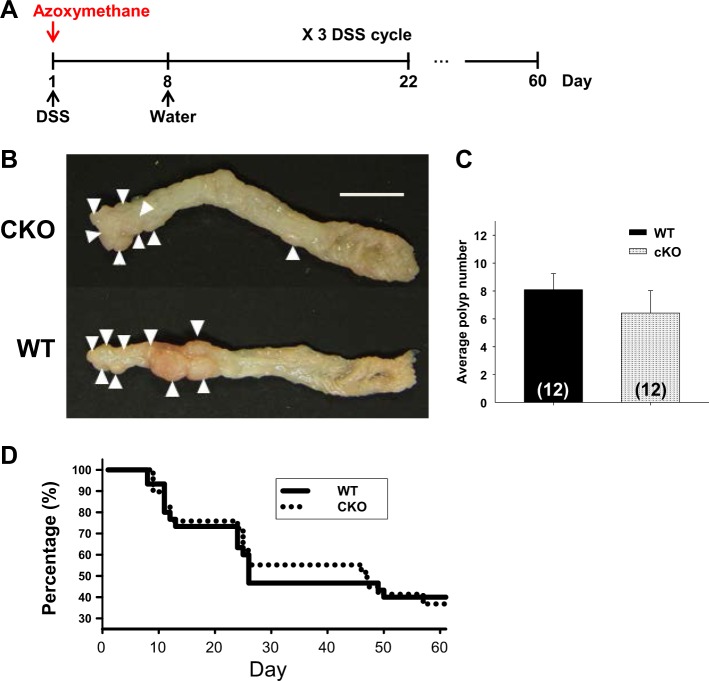
*Ano1*-deficient colons were not susceptible to colorectal cancer. **a** Schematic protocol for the model of colorectal cancer induced by azoxymethane and DSS. After the intraperitoneal injection of azoxymethane (10 mg/kg), 2% DSS was given in drinking water for 8 days. The mice then recovered for 14 days. The DSS treatment cycle was repeated three times, and the mice were sacrificed on day 60. **b** Colons of *Ano1*^fl/fl^ (WT) and *Cdx2-Ano1*^fl/fl^ (CKO) mice after treatment with azoxymethane and DSS. Polyps are indicated by arrowheads. **c**, **d** Number of polyps (**c**) and incidence of death (**d**) in WT (*n* = 29) and CKO (*n* = 30) mice after treatment with azoxymethane plus DSS

Next, we developed a model of colorectal cancer in the colon of *Cdx2-Ano1*^fl/fl^ mice to determine whether the mutant mice had a greater susceptibility to colorectal cancer. Colorectal cancer was induced by the intraperitoneal injection of azoxymethane (10 mg/kg) at the beginning of the experiment, followed by three DSS cycles (Fig. 7a). Each cycle consisted of DSS treatment for 8 days followed by treatment with normal water for 14 days^41^. Pathological analysis was conducted on day 60 (Fig. 7a). Polyps (6.42 ± 1.6; *n* = 12) were found primarily in the proximal colons of the *Cdx2-Ano1*^fl/fl^ mice treated with azoxymethane and DSS (Fig. 7b, c). The number of polyps was comparable to that observed in the colons of the *Ano1*^fl/fl^ mice treated with azoxymethane and DSS (8.08 ± 1.16; *n* = 12) (Fig. 7c). Moreover, the incidence of death of the conditional knockout mice was not different from that of the wild-type mice (Fig. 7d). Thus, these results indicate that the ablation of *Ano1* in the colon does not affect the development of colonic polyps.

## Discussion

The intestinal secretion of Cl^−^ is thought to be necessary for the protection of the epithelia of the gut and is well controlled by intrinsic, hormonal, and neural factors. Its excessive or deficient function leads to pathologic conditions that are sometimes lethal^1^. The intestinal secretion of Cl^−^ is thought to be mediated by CFTR and CaCCs present on the apical surface of the intestine^5^. The secretion of Cl^−^ mediated by CFTR is important for many examples of hormone-induced and toxin-induced fluid secretion. However, our understanding of the role of CaCCs as a source of Cl^−^ and fluid secretion in the intestine remains incomplete. In this study of *Ano1-*deficient mice with a conditional knockout in the epithelial cells of the colon, we proved that ANO1 also contributes to the secretion of intestinal fluid. Cl^−^ and fluid secretion were reduced in the intestines of the *Ano1*-deficient mice. Mild edema was observed under basal conditions when ANO1 was ablated. *Ano1* disruption in the colon led to much greater susceptibility to colitis. Furthermore, the cAMP-dependent secretion of Cl^−^ and cholera toxin-induced fluid secretion were also significantly reduced in the intestines of *Ano1*-deficient mice, suggesting that cholera toxin and cAMP signal by means of CaCCs in addition to CFTR. Thus, this study provides evidence for the roles of CaCCs in proximal small intestinal and proximal colonic Cl secretion in mice.

The Cl^−^ currents induced by the application of forskolin and IBMX were markedly reduced in colonocytes from *Ano1*-deficient mice (Fig. 4). This finding is surprising because it has long been believed that cAMP-induced currents are only mediated by CFTR^9^. The reduction in cAMP-induced Cl^−^ currents was not a result of the downregulation of CFTR because its protein level was not changed by the deletion of *Ano1* (Fig. 4). Indeed, the transepithelial currents evoked by the application of forskolin and IBMX were markedly suppressed by an ANO1 antagonist, MONNA (Fig. 4). Importantly, the cholera toxin-treated intestine secreted significantly less fluid in the jejunum in *Ano1*-deficient mice, which further supports a role for *Ano1* in cAMP-induced intestinal secretion. The activation of CFTR is thought to be one of the main causes of cholera-induced diarrhea. A synergistic activation mechanism between CFTR and CaCCs has been reported^54^, which is consistent with this study. Forskolin was also found to evoke a calcium response that causes Cl^–^ secretion^35^. Thus, it seems likely that an increase in intracellular cAMP induced by toxins or another pathologic condition may activate ANO1 as well as CFTR.

Electrophysiological studies have suggested the presence of Ca^2+^-activated secretion of Cl^−^ or currents in the small intestines or colon^5^. Ca^2+^-dependent Cl^−^ conductance has been observed in a human colonic epithelial cell line, T84 cells^16,17,27^. Carbachol induces Cl^−^ secretion in the small intestine of *Cftr*^−/−^ mice^15,55^. In addition, Bronsveld and colleagues observed the carbachol-induced secretion of Cl^−^ in the intestines of patients with cystic fibrosis^56^. Thus, these studies support the role of CaCCs in fluid secretion in the intestines. In contrast, the intestinal epithelia of patients with cystic fibrosis and the intestines of *Cftr*^−/−^ mice do not secrete Cl^−^ in response to a Ca^2+^ ionophore, which argues against the functional presence of CaCCs^36,57^. Thus, whether CaCCs are involved in intestinal fluid secretion has not been fully resolved. ANO1 is a type of CaCC activated by intracellular calcium^19–21^. Thus, the expression of ANO1 in the intestines supports the role of CaCCs in Cl^−^ secretion in the intestines. Previous studies with PCR revealed high expression of ANO1 in the colon but weaker expression in segments of the small intestine^32,58^. Quantitative PCR analysis of different regions of the GI tract revealed that the highest expression of ANO1 is found in the distal colon, with weaker expression in the duodenum and ileum, but not in the jejunum^58^. Similarly, when determined by RT-PCR, Schreiber and colleagues found high expression of ANO1 in the proximal and distal colon and weak expression of ANO1 in the ileum, but not in the jejunum^32^. In the present study, we also found high expression of ANO1 in isolated epithelial cells of the proximal and distal colon but weaker expression in the jejunum and ileum. Despite some differences in the expression patterns of ANO1 in various segments of the intestines, high expression of ANO1 in the colon is largely consistent. Thus, the expression of ANO1, a type of CaCC, in the intestine further suggests the role of ANO1 in Cl^−^ secretion.

Models for a chloride secretory mechanism in the intestine were suggested previously and were considered to be similar to those found in other secretory transport epithelia^5,59,60^. According to these models, to transport Cl^−^ from the serosal to the luminal side, Cl^-^ is taken up from the serosal side by Na–K–2Cl cotransporter 1 (NKCC1) in the basolateral membrane of epithelial cells, which accumulates Cl^−^ in the cell. Because NKCC1 also pumps Na+ and K+ into the cell, potassium channels in the basolateral membrane need to be active in recycling K+ out of the cell to prevent depolarization. As Cl^−^ accumulates in the cell, Cl^−^ needs to exit the cell via Cl^−^ channels present in the apical membrane. These channels are thought to be CFTR and CaCCs. This study largely supports this model of Cl^−^ secretion. ANO1 immunoreactivity was found largely in the apical membranes of the jejunum and the colon, as it colocalized with phalloidin, a marker of the apical membrane^28^ (Fig. 3). In addition, ANO1 immunoreactivity was rarely colocalized with β-catenin, a marker of the basolateral membrane^61^. The presence of ANO1 immunoreactivity in the apical membrane was also observed by Yu and colleagues^28^. In contrast, Schreiber and colleagues suggested a different model in which ANO1 is present in the basolateral membrane, largely based on the fact that the carbachol-induced currents are reduced by the basolateral application of its blockers^32^. According to their model, Cl^−^ enters epithelial cells via ANO1 from the basolateral membrane when stimulated by carbachol and exits through the CFTR channel in the apical membrane.

In summary, ANO1 contributes to the secretion of Cl^−^ in the murine colon. The disruption of *Ano1* in the intestine causes impaired secretion of Cl^−^ and elicits mild edema. Because ANO1 appears to partially mediate cAMP-dependent fluid secretion, ANO1 blockers may be advantageous in the treatment of enterotoxin-related diarrhea. Additionally, because Cl^−^ secretion through CaCCs is considered to be an alternative pathway to compensate for the pathologic effects of cystic fibrosis, the findings of this study further suggest the suitability of ANO1 as a promising target for the development of drugs to compensate for CFTR malfunction in patients with cystic fibrosis. During the revision of this paper, Benedetto and colleagues reported that ANO1 is essential for the proper membrane function of CFTR, which further supports the functional link between ANO1 and CFTR^62^.

## Supplementary information

Supplementary Figure 1

Supplementary Figure 2

Supplementary Figure 3
