# Supplementary figures and images for: Anoctamin 1/TMEM16A controls intestinal Cl− secretion induced by carbachol and cholera toxin
Source: Exp Mol Med. 2019 Aug 5;51(8):91. doi: 10.1038/s12276-019-0287-2 (PMC6802608; doi:10.1038/s12276-019-0287-2)

## Slide 1
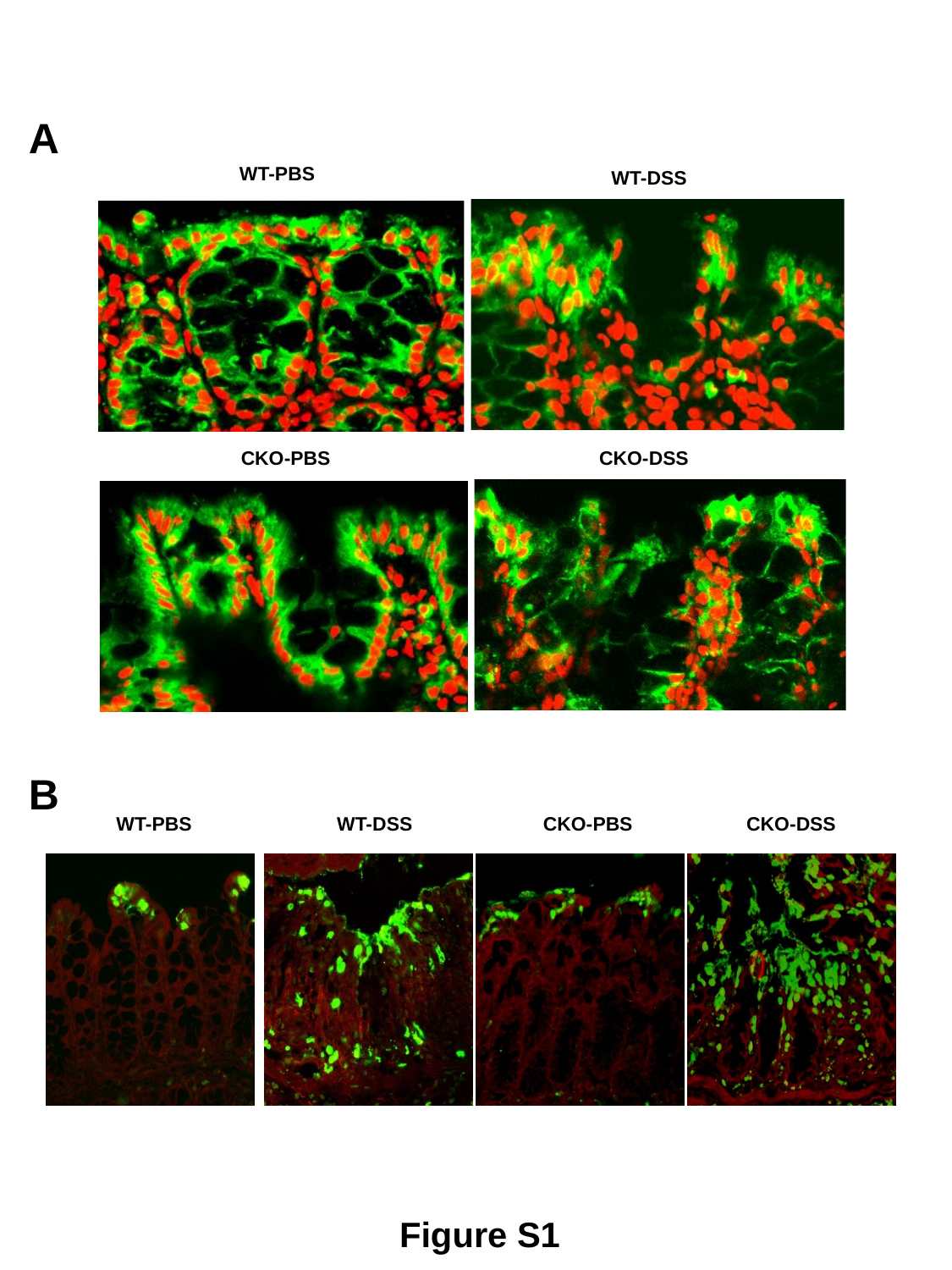

A
WT-PBS
WT-DSS
CKO-PBS
CKO-DSS
B
WT-PBS
WT-DSS
CKO-PBS
CKO-DSS
Figure S1

Supplement: Supplementary file 1 — Supplementary Figure 1 [file 12276_2019_287_MOESM1_ESM.pptx]

## Slide 1
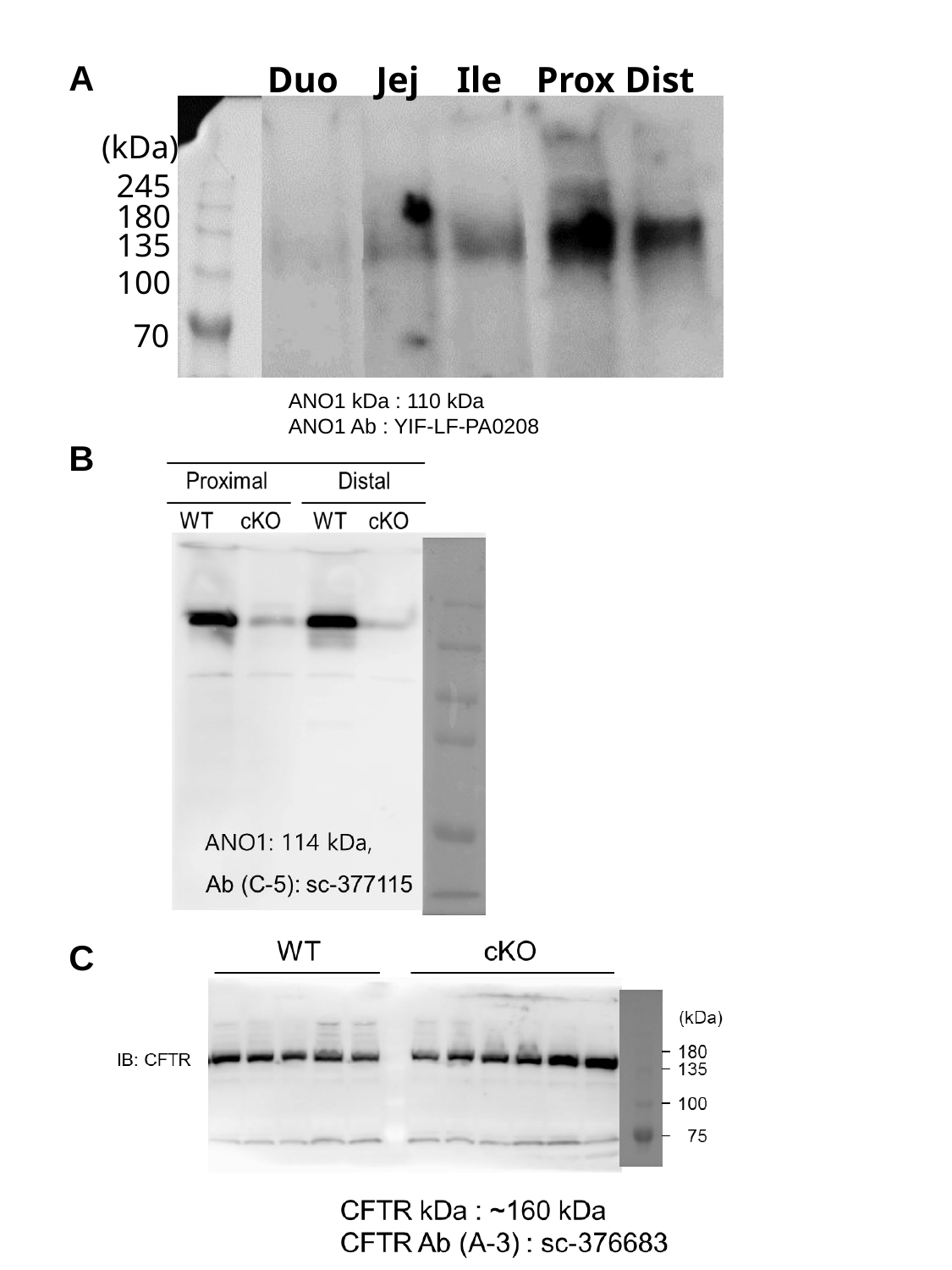

A
Duo
Jej
Ile
Prox
Dist
(kDa)
245
180
135
100
70
ANO1 kDa : 110 kDa
ANO1 Ab : YIF-LF-PA0208
B
C

Supplement: Supplementary file 3 — Supplementary Figure 3 [file 12276_2019_287_MOESM3_ESM.pptx]
