# Supplementary material for: Anoctamin 1/TMEM16A controls intestinal Cl− secretion induced by carbachol and cholera toxin
Source: Exp Mol Med. 2019 Aug 5;51(8):91. doi: 10.1038/s12276-019-0287-2 (PMC6802608; doi:10.1038/s12276-019-0287-2)
Supplement: Supplementary file 2 — Supplementary Figure 2 [file 12276_2019_287_MOESM2_ESM.pptx]

## Slide 1
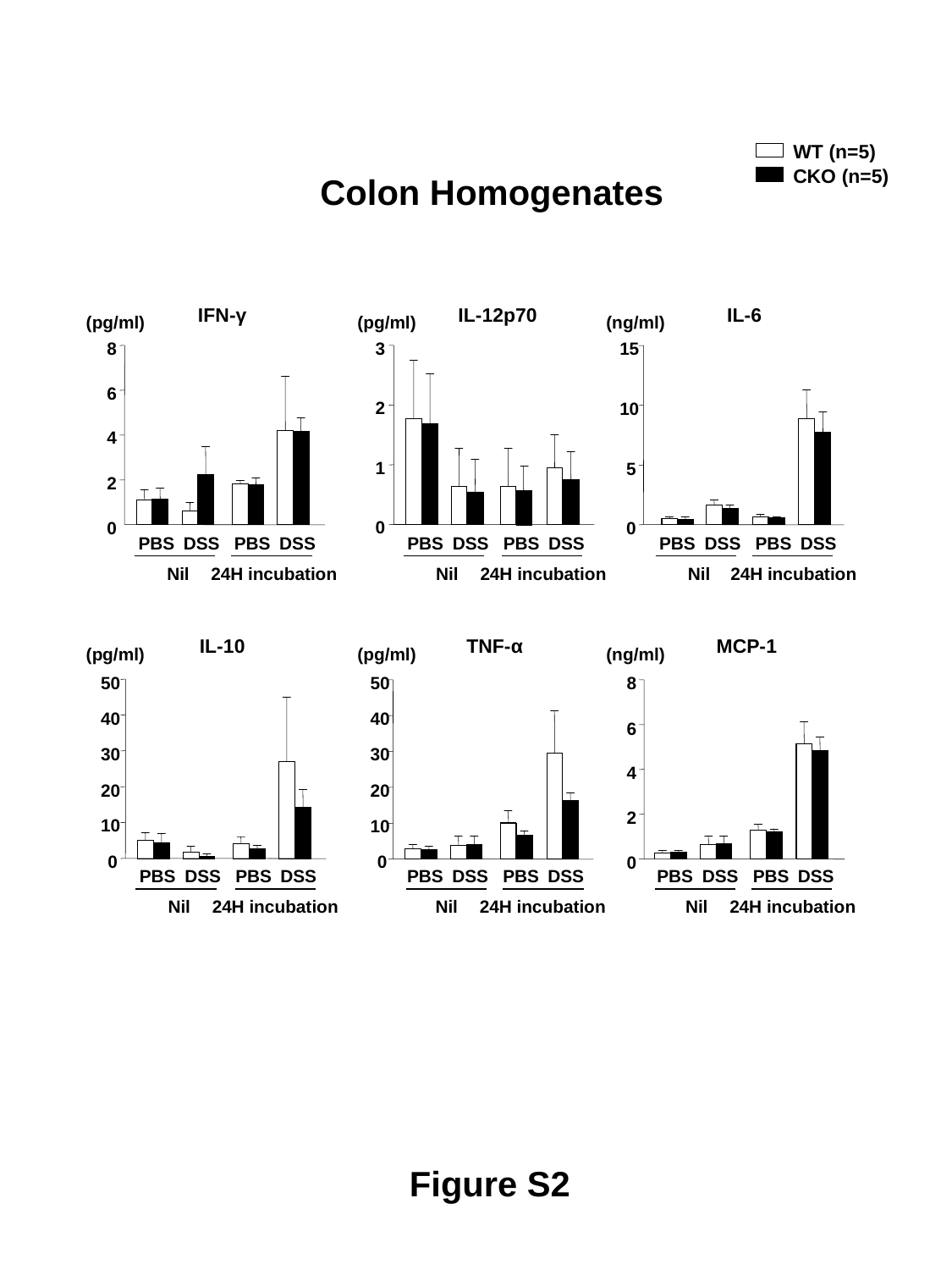

WT (n=5)
CKO (n=5)
Colon Homogenates
IFN-γ
IL-12p70
IL-6
(pg/ml)
(pg/ml)
(ng/ml)
3
8
15
6
2
10
4
1
5
2
0
0
0
PBS
DSS
PBS
DSS
PBS
DSS
PBS
DSS
PBS
DSS
PBS
DSS
Nil
24H incubation
Nil
24H incubation
Nil
24H incubation
IL-10
TNF-α
MCP-1
(pg/ml)
(pg/ml)
(ng/ml)
8
50
50
40
40
6
30
30
4
20
20
2
10
10
0
0
0
PBS
DSS
PBS
DSS
PBS
DSS
PBS
DSS
PBS
DSS
PBS
DSS
Nil
24H incubation
Nil
24H incubation
Nil
24H incubation
Figure S2
